# Safety level assessment of shield tunneling in water rich sandy pebble strata with large particle size

**DOI:** 10.1038/s41598-023-30718-5

**Published:** 2023-03-08

**Authors:** Peiyi Yao, Ziwang Yu, Yan Lv, Bin Shi, Yuanyuan He, Hong Wang, Danna Liu, Shengda Wei

**Affiliations:** 1 Construction Engineering College, Jilin University, Changchun, 130026 Jilin China; 2 College of Civil Engineering, Guizhou University, Guiyang, 550025 Guizhou China; 3 China Communication North Road and Bridge Co., Ltd., Beijing, 100024 China

**Keywords:** Civil engineering, Geology

## Abstract

Shield tunneling method is widely used in urban metro construction. The construction stability is closely related to the engineering geological conditions. Sandy pebble strata have a loose structure and low cohesion, resulting in great engineering-induced stratigraphic disturbance. Meanwhile, the high water-abundance and strong permeability are extremely detrimental to construction safety. It is of great significance to evaluate the dangerousness of shield tunneling in water-rich pebble strata with large particle size. In this paper, risk assessment of engineering practice is carried through with Chengdu metro project in China as a case study. Referring to the special engineering situations and assessment workload, seven evaluation indices, including compressive strength of pebble layer, boulder volume content, permeability coefficient, groundwater depth, grouting pressure, tunneling speed and tunnel buried depth are selected to establish an evaluation system. A complete risk assessment framework is established based on the cloud model, AHP and entropy weight method. Further, the measured surface settlement is taken as the risk degree characterization to verify the results. This study can provide reference for method selection and evaluation system establishment in the risk assessment of shield tunnel construction in water-rich sandy pebble strata, and contribute to proposing safety management in similar engineering projects.

## Introduction

In order to make transportation and life more convenient, urban metro construction has developed rapidly in recent years. The open excavation method, shallow buried excavation method and shield method are all the main methods adopted to the urban metro construction^1^. Among them, the shield method is the most widely chosen, with the advantages of short construction period and high safety performance. It is commonly used to excavate soil and sandy soil surrounding rock in tunnel by using a shield machine. The basic principle of its work is to excavate the soil along the design axis of the tunnel with a shield machine and move forward. The shield shell is used as temporary support during tunnel excavation, and then segments are assembled under the protection of shield shell to form permanent lining^2^. The construction and excavation, however, will inevitably lead to stratum disturbance. Harsh engineering geological conditions have a great negative impact on the stable construction of shield tunneling, which may lead to surface collapse, deformation and inclination of buildings around the surface, and even endanger the construction safety. It is of great significance to perform safety level assessment, identify dangerous sections in advance and take care of protection.

During the shield tunneling process, if the shield machine suddenly malfunctions, such as being stuck or the cutting tool becomes unstable, it will pose a great threat to construction safety^3^. In order to ensure the stability of the construction, scholars have conducted studies related to the initiation of shield machine failures. Some of the representative ones are energy consumption and life prediction of shield cutter drive^4,5^, influence analysis of shield pitch angle change on construction safety^6^, mechanism investigation of pore variation characteristics of shield excavation face^7^, and effect study of rock extrusion interaction on formation pressure and shield machine failure induction^8^. In addition to the above factors, the site engineering geological condition such as the nature of excavation soil have an impact that cannot be ignored in the actual engineering project. Shield tunneling in water-rich sandy pebble strata should especially paid attention to construction safety issues. That is because the sandy pebble strata have larger particle size and pore ratio, stronger water-rich and permeability, lower bearing capacity and stability than others. When the shield machine operates in large-diameter pebbles, the wear and damage of cutting tools will be aggravated. Besides, the possibility of large size pebbles being trapped in the soil compartment will increase relatively, leading to soil jamming and shield tunneling failure^9^. All of the above are huge challenges, and the negative effect of groundwater adds to the difficulty of construction. However, in contrast to the previous tunneling mostly in sandy or clay soils^10^, little attention has been paid to carry out the construction risk evaluation under the special condition of water-rich sandy pebble strata.

Throughout the international research landscape, there has been quite abundant studies aim at the topic of safety in shield tunnel construction. These studies cover a wide range of research perspectives, including the specific hazards response to engineering such as ground settlement and water inrush^11,12^, influencing factors and safety analysis of construction stability^13,14^, prediction of shield moving trajectory under complex geological conditions^15^, the development of outdoor monitoring techniques^16^ and indoor multi-dimensional modeling^17^, as well as the innovation of evaluation methods for construction risk^18,19^, etc. Among these numerous risk assessment methods, the cloud model is widely used and has achieved good results. The cloud model is a mathematical theory established on the basic of probability theory and fuzzy mathematics, which is used for realizing the transformation between qualitative concepts and quantitative values^20^. It has the unique advantage of considering both randomness and fuzziness in the evaluation, which coincide with the characteristics of the construction risk occurrence^21,22^. Liu et al.^23^ evaluated the stability of shield tunnel excavation face through cloud model theory. Combined the cloud model with Bayesian network, Zhang et al.^24^ established an analytical model for predicting the damage risk of other existing buildings caused by tunnel construction. By introducing the variable weight function into the cloud model, Lin et al.^25^ realized the establishment of a new quantitative evaluation method of tunnel water inrush disaster. All the above studies indicated that the cloud model has wide and good applicability. Meanwhile, the cloud model can be used in combination with other methods to achieve better effect.

The construction risks are diverse and influence factors are highly uncertain. The implementation of the classical cloud model method is based on the assumption that the evaluation indicators obey a normal distribution. But this is not always the case in practice, and relative influence between indicators will transmit to the accuracy of evaluation results^26^. It is particularly important to establish the evaluation system by considering various factors and assign reasonable indicator weights. Analytic hierarchy process (AHP) and entropy weight method (EWM) are typical subjective and objective weighting method respectively, and the combination of the two methods can not only retain the expert experience but also ensure data support^27^. However, how to design the allocation in the combination of subjective and objective weights is a puzzle that requires careful consideration.

In view of this, this paper established a complete risk assessment framework for shield tunnel construction in water-rich sandy pebble strata by fully utilizing the cloud model, AHP and EWM. In this method, the forward cloud model is used to obtain the certainty of safety level evaluation. In order to provide more sensible weights, the reverse cloud model is applied to process the allocation of the subjective and objective weights obtained by AHP and EWM. In this way, the advantages of the cloud model with randomness and fuzziness can be fully exploited, and an excellent solution to the weight assignment is provided. Based on the Chengdu Metro project in China, the evaluation framework was applied to evaluate the safety level of shield tunneling in large particle size water-rich sandy pebble strata. Further, the magnitude of surface deformation monitored by the site was taken as an accurate reference to verify the evaluation results.

## Methodology

### Cloud model

The cloud model is a transformation model proposed by Li et al.^20^, which based on the uncertainty of qualitative concepts and quantitative values. The realization of cloud model depends on probability theory and fuzzy mathematics. It mainly discusses the relationship between fuzziness and randomness of things. The cloud model has three cloud digital eigenvalues, which are *E*_*x*_*, E*_*n*_ and *H*_*e*_*. E*_*x*_, the most typical value, is the expected value of cloud in the domain space. Equation (1) is the calculation formula of *E*_*x*_ in the cloud model, in which *a* and *b* are the minimum boundary value and maximum boundary value of the evaluation index corresponding to the risk level evaluation criteria. *E*_*n*_ is a measure of uncertainty and randomness of qualitative concepts, which can be solved according to Eq. (2)^28^. *H*_*e*_ is the hyper entropy, which measures the uncertainty of entropy and reflects the correlation between randomness and fuzziness of research objects. The value of *H*_*e*_ can be adjusted according to the fuzzy threshold of the index, just as shown in Eq. (3). In order to avoid atomization of cloud droplet distribution, *k* is usually taken as 0.1^29^.

1 $$ E_{x} = \frac{a + b}{2} $$

2 $$ E_{n} = \frac{b - a}{{2.355}} $$

3 $$ H_{e} = k \times E_{n} $$

Cloud generator is a tool to establish the relationship between the qualitative and the quantitative. Inputting *E*_*x*_*, E*_*n*_*, H*_*e*_ and the number of cloud droplets into the forward normal cloud generator to calculate the cloud droplets and obtain the certainty degree, as shown in Eq. (4), in which *x*_*i*_ is a normal random number with expectation *E*_*x*_ and variance *E*_*n*_, *y*_*i*_ is a normal random number with expectation *E*_*n*_ and variance *H*_*e*_. On the contrary, if several cloud droplets are input, the three eigenvalues of the cloud can also be obtained through the reverse cloud generator^30^. The numerical characteristics of the reverse cloud generator are expressed as Eqs. (5)–(8), in which *E*_*x*_^*’*^ is the sample mean, *S*^*2*^ is the sample variance, *E*_*n*_^*’*^ is the entropy, *H*_*e*_^*’*^ is the hyper entropy and *n* is the number of cloud droplets.

4 $$ \mu_{i} = exp\left[ { - \frac{{\left( {x_{i} - E_{x} } \right)^{2} }}{{2\left( {y_{i} } \right)^{2} }}} \right]{ } $$

5 $$ E_{x}^{^{\prime}} = \frac{{\mathop \sum \nolimits_{i = 1}^{n} x_{i} }}{n} $$

6 $$ S^{2} = \frac{{\mathop \sum \nolimits_{i = 1}^{n} \left( {x_{i} - E_{x}^{^{\prime}} } \right)^{2} }}{n - 1} $$

7 $$ E_{n}^{^{\prime}} = \frac{{\sqrt {\frac{\pi }{2}} \times \mathop \sum \nolimits_{i = 1}^{n} \left| {x_{i} - E_{x} {^{\prime}}} \right|}}{n} $$

8 $$ H_{e}^{^{\prime}} = \sqrt {S^{2} - E_{n}^{{^{\prime}2}} } $$

### Analytic hierarchy process (AHP)

Analytic hierarchy process (AHP) was proposed by American operational research expert Saaty, it constructs a progressive hierarchy structure by the relationships among the factors in the system. Due to the simplicity, convenience and universality of AHP, it has attracted the attention of scholars in various fields and has been revised and improved^31–35^. Based on a criterion in the upper layer, compare the importance of each element of the same layer to construct a judgment matrix. And then get the relative weight of a certain element to the criterion. Finally, the subjective weights are calculated according to all the elements and criteria. The weight calculation process of AHP is shown in Eqs. (9)–(15), that proposed by Saaty^36^. First of all, construct the judgment matrix **R** as shown in Eq. (9). And then compare the relative importance of index *i* and *j*. If index *i* is more important, **r**_**ij**_ is taken one. If index *i* and index* j* have the same importance, then **r**_**ij**_ is taken zero. If the index *j* is more important, **r**_**ij**_ is taken minus one. Subsequently, calculate the optimal transfer matrix **B** and consistency judgment matrix **C**. The elements in matrix **B** and matrix **C** are shown as Eq. (10) and Eq. (11). In the end, calculate the eigenvector of judgment matrix **C** by Eq. (12). And the weights can be obtained through normalization, as shown in Eqs. (13)–(15)*.*

9 $$\mathbf{R}={\left({\mathbf{r}}_{\mathbf{i}\mathbf{j}}\right)}_{n\times n}$$

10 $${\mathbf{b}}_{\mathbf{i}\mathbf{j}}=\frac{1}{n}\sum_{k=1}^{n}\left({\mathbf{r}}_{\mathbf{i}\mathbf{k}}+{\mathbf{r}}_{\mathbf{k}\mathbf{j}}\right)$$

11 $${\mathbf{c}}_{\mathbf{i}\mathbf{j}}=\mathrm{exp}{\mathbf{b}}_{\mathbf{i}\mathbf{j}}$$

12 $${P}_{i}=\prod_{j=1}^{n}{\mathbf{c}}_{\mathbf{i}\mathbf{j}}$$

13 $$\overline{{w}_{ai}}=\sqrt[n]{{P}_{i}}$$

14 $$\overline{{w}_{a}}={\left[\overline{{w}_{a1}},\overline{{w}_{a2}},\cdots ,\overline{{w}_{an}}\right]}^{T}$$

15 $${w}_{a}={\left[{w}_{a1},{w}_{a2},\cdots ,{w}_{an}\right]}^{T}$$

### Entropy weight method (EWM)

In the evaluation system, the amount of information carried by each index is different. The concept of information entropy was put forward in 1948, which was used to describe the degree of chaos of a system and specifically represent the degree of dispersion of an indicator. The basic principle of entropy weight method (EWM) is to determine the index weight according to the amount of information carried by the index. The index with a large amount of information has a greater impact on the evaluation system, so the entropy weight and the index weight are also relatively large. Otherwise, the index weight is small^37^. As an objective weighting method, EWM is usually not used alone in the relevant studies of risk assessment, but combined with attribute recognition theory^22^, unascertained measure theory^38^, set pair theory^39^and other methods. The weight calculation process of EWM is shown in Eqs. (16)–(19), that provided by Zhang et al.^37^. Firstly, calculate the proportion of the instance *i* under the evaluation index* j* by using Eq. (16) and Eq. (17), in which **X** is the corresponding matrix of sample data values,* m* is the number of instances and *n* is the number of evaluation indices. Subsequently, calculate the index information entropy, according to Eq. (18). At last, the index weights can be obtained by Eq. (19).

16 $${P}_{ij}=\frac{{\mathbf{x}}_{\mathbf{i}\mathbf{j}}}{\sum_{i=1}^{n}\left({\mathbf{x}}_{\mathbf{i}\mathbf{j}}\right)},j=\mathrm{1,2},\cdots ,n$$

17 $$\mathbf{X}=({{\mathbf{x}}_{\mathbf{i}\mathbf{j}}) }_{m\times n}$$

18 $${E}_{j}=-\frac{1}{\mathit{ln}n}\sum_{i=1}^{n}\left({P}_{ij}\times \mathrm{ln}{P}_{ij}\right)$$

19 $${w}_{ej}=\frac{exp\left[\sum_{k=1}^{n}\left({E}_{k}\right)+1-{E}_{j}\right]-exp{E}_{j}}{\sum_{l=1}^{n}\left\{exp\left[\sum_{k=1}^{n}\left({E}_{k}\right)+1-{E}_{l}\right]-exp{E}_{l}\right\}}$$

### Establishment of evaluation process

The combination of cloud model and classical weight assignment methods has been a key tool to deal with uncertainty research problems such as risk assessment. The cloud model has generally been used to solve the certainty degree, combined with weight assignments to obtain accurate assessment results^22,25^. The weight assignment methods can generally be classified into three categories, including subjective weighting method, objective weighting method and combination weighting method. The combination weighting method takes into account both subjectivity and objectivity, which makes the weighting process more rational. At this stage, the percentages of subjective and objective weights are generally determined based on expert experience^40^. The cloud model, as an important tool that can deal with the linkage and unification between qualitative and quantitative issues, can solve the defects of the existing weight setting methods that cannot reflect cognitive uncertainty. Treating the weight assignment of evaluation index as a fuzzy stochastic problem, the gradual optimization of the weight solving process can be achieved with the help of the cloud generator.

**Figure 1:**
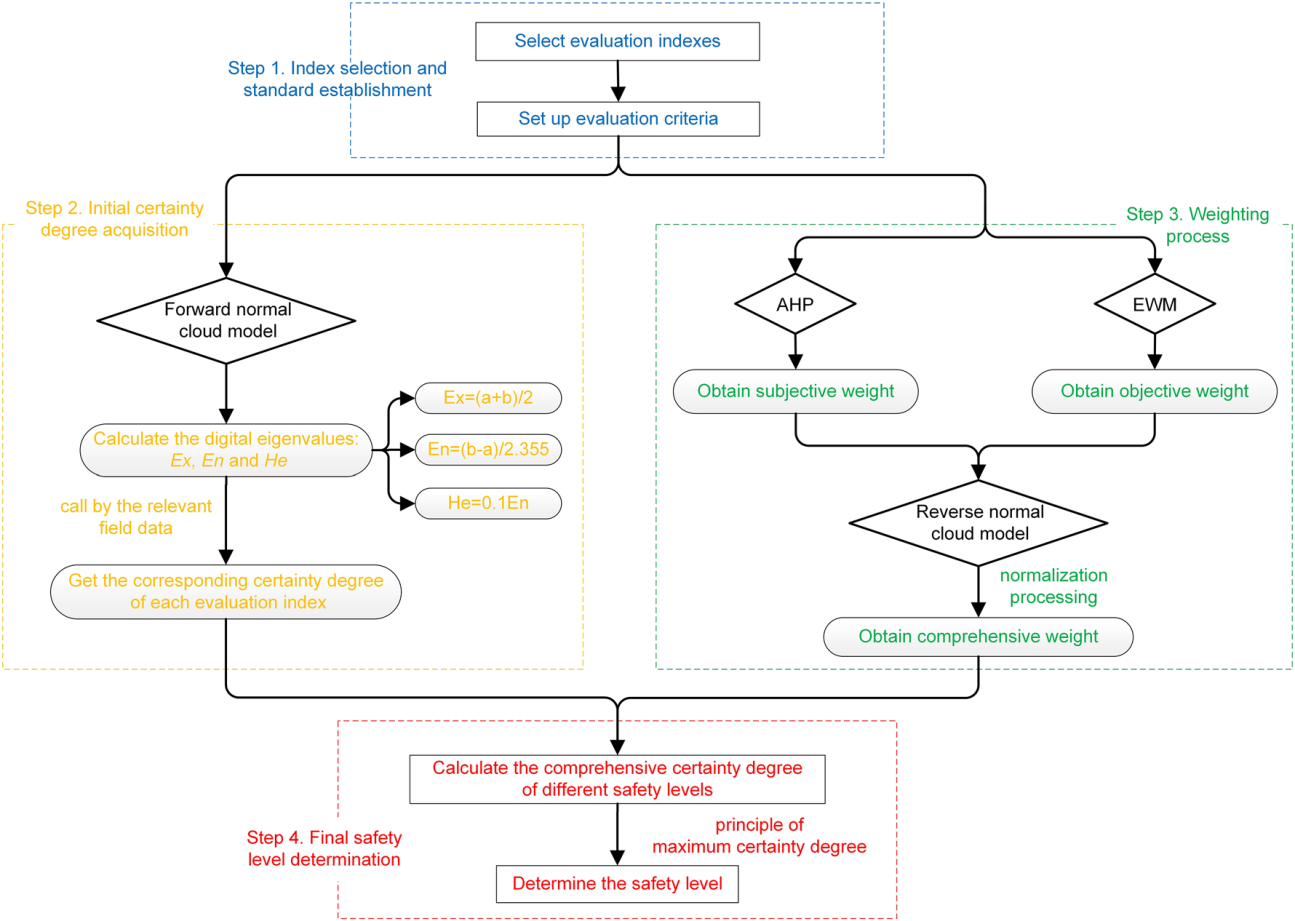
Evaluation flow chart.

On the basis of the comprehensive consideration of the accuracy and simplicity of evaluation work, cloud model, AHP and EWM are combined to establish the following risk assessment process. The forward cloud model is used to achieve membership output of grade evaluation. AHP and EWM are used to obtain the subjective and objective weights respectively. The reverse cloud model is used to solve for the comprehensive weight. The final evaluation result is obtained by multiplying the comprehensive weight by the initial certainty degree. The composite model is clear in principle and simple in application, which can maximize the advantages of each approach and enabling better solutions to the research problem. The evaluation flow chart is shown as Fig. 1. The evaluation process can be divided into four steps basically: (1) index selection and standard establishment; (2) initial certainty degree acquisition; (3) weighting process; and (4) final safety level determination, which are respectively corresponding to the blue, yellow, green and red dashed boxes in Fig. 1.

***Step 1: Index selection and standard establishment***

The primary requirement for risk assessment is to get a comprehensive understanding of the evaluation indices. The selection of evaluation indices and construction of evaluation system are the beginning and basis for the whole evaluation. Under the premise of limited data availability, risk induced indices should be considered comprehensively, and factors with low correlation and high redundancy should be eliminated. After the completion of the index evaluation system, the establishment of appropriate grading standard is of great significance affecting the accuracy of the results. Combined with the actual situation of the project, formulate the evaluation standard from safety to danger gradually and set the reasonable grading span.

***Step 2: Initial certainty degree acquisition***

In the step 2, the cloud model is used to calculate the certainty degree of evaluation index according to the evaluation criteria obtained in the previous step. According to Eqs. (1)–(3), the cloud digital eigenvalues, which are *E*_*x*_, *E*_*n*_ and *H*_*e*_, of each safety level corresponding to the evaluation indices are obtained, and the cloud model is generated by forward normal cloud generator. Collect the relevant data of project, and put the values of each evaluation index into the cloud model. Then, the corresponding certainty degree of each evaluation index with a stable tendency can be obtained by Eq. (4).

***Step 3: Weighting process***

The step 3 is to use AHP, EWM and reverse cloud model to obtain the comprehensive weight of indices, and the specific process is as follows. Firstly, the subjective and objective weights are calculated by AHP and EMW, using Eqs. (9)–(15) and Eqs. (16)–(19) respectively. Then, these two types of weights are regarded as a group of cloud droplets surrounding the real weight and used to generate the cloud by making random complements to the cloud drops^41^. And the expectation of the cloud drops distribution can be solved to obtain the composite cloud weight by the reverse cloud model, using Eqs. (5)–(8). Later, normalize the composite cloud weight to get the comprehensive weight of each index with Eq. (20), in which *λ*_*i*_ is the comprehensive weight, *w*_*i*_ is the composite cloud weight and *m* is the number of indices.

20 $$ \lambda_{i} = \frac{{w_{i} { }}}{{\mathop \sum \nolimits_{i = 1}^{m} w}} $$

***Step 4: Final safety level determination***

**Figure 2:**
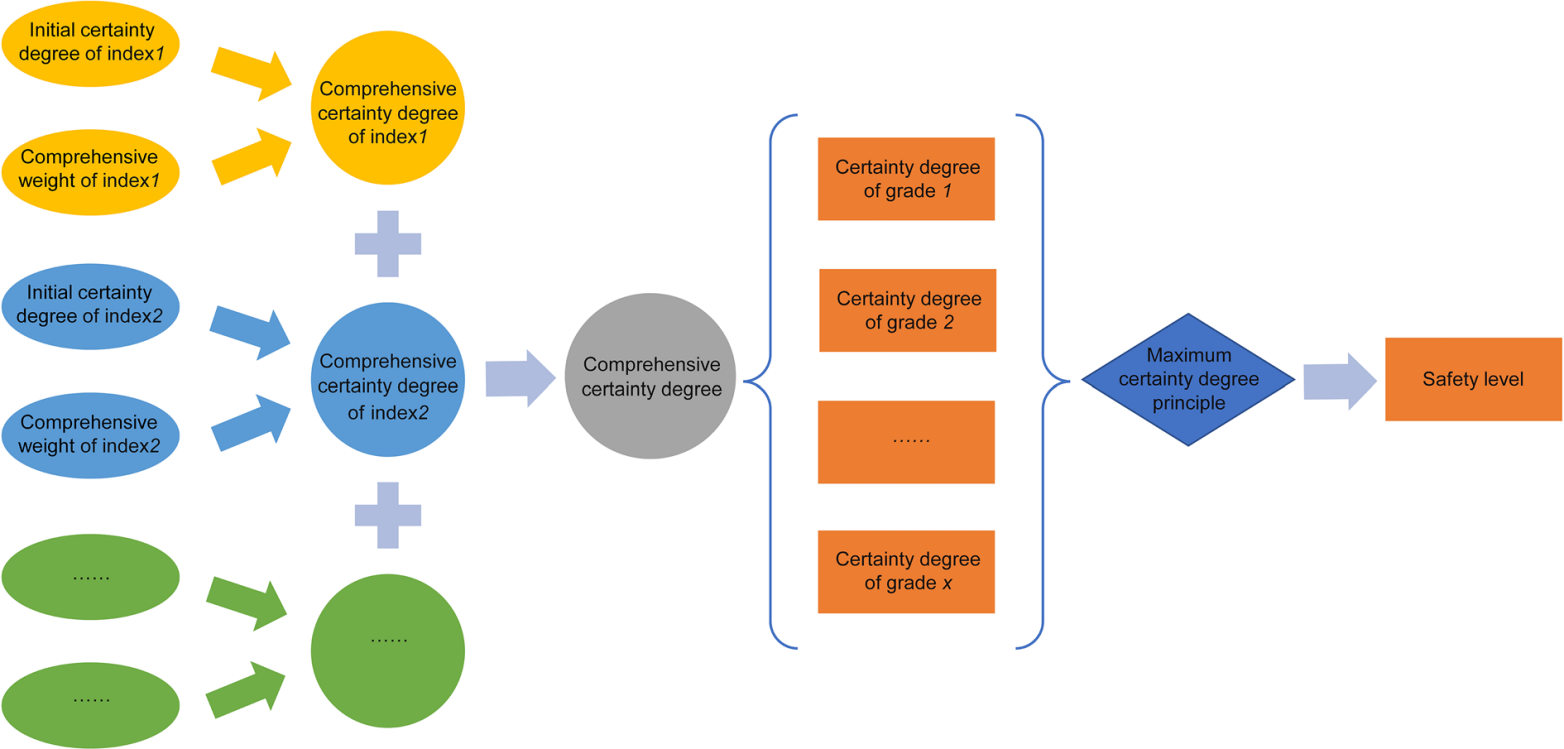
Determination of final safety level.

In the step 4, calculate the comprehensive certainty of different safety levels and determine the final safety level according to the principle of maximum certainty degree. The comprehensive certainty degree is obtained by adding the weighted certainty degree of each evaluation index, as shown in Eq. (21), in which *U*_*x*_ is the comprehensive certainty degree of the safety level *x* and *U*_*xi*_ is the certainty calculated by the evaluation index *i*. Figure 2 shows the whole process of final safety level determination.

21 $$ U_{x} = \mathop \sum \limits_{i = 1}^{n} U_{xi} {*}\lambda_{i} { },{ }x = 1,2,3, \ldots ,n $$

## Engineering application

### General situation of project

**Figure 3:**
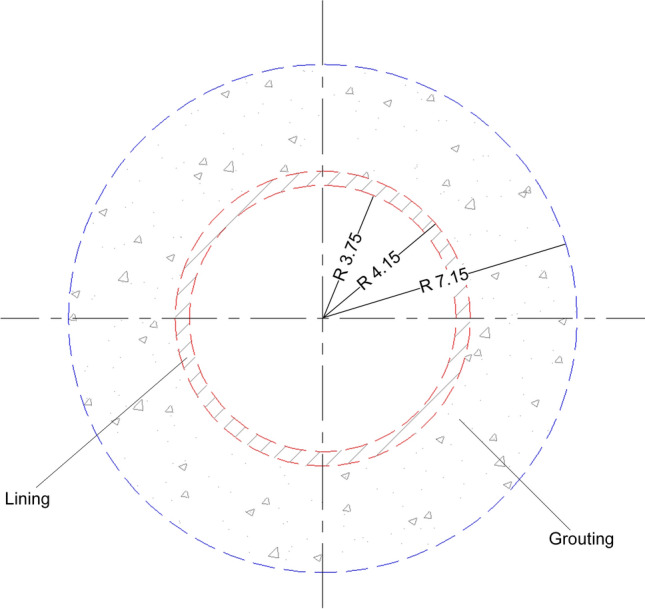
Cross-sectional diagram of the shield tunnel.

**Figure 4:**
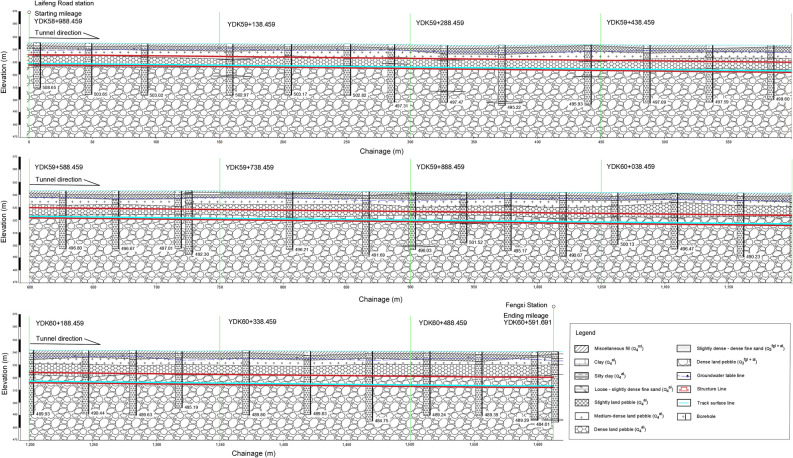
Geological profile of the evaluated tunnel section.

Taking the water-rich sandy pebble strata interval of the Chengdu Metro project as a case study, the evaluation process established previously is applied to evaluate the safety level of construction. The assessment interzone tunnel section is from Laifeng Road Station to Fengxi Station, with a total length of about 1603.2 m (YDK58 + 988.459 − YDK60 + 591.691). The small mileage is the shield originating well, while the large mileage is the shield receiving well. The tunnel outer diameter is 8.3 m, and the effective inner diameter of the shield is 7.5 m. The total lining thickness is 0.4 m and the grouting thickness is 3 m. The cross-sectional diagram of the shield tunnel is shown in Fig. 3. The buried depth of the roof of the interzone tunnel is 9.57–20.14 m, and the buried depth of the floor is 17.57–28.14 m. A total of 37 boreholes were drilled at depths ranging from 45-55 m every 40 m-60 m along the tunnel, which provided information on the relevant geological conditions in the study area. All the sites are covered by quaternary (Q) strata, and the surface is mostly covered by artificial fill (Q_4_^ml^). The artificial fill follows the sand and pebble soil in the Holocene alluvial (Q_4_^al^), the upper Pleistocene glacial water deposition and alluvial (Q_3_^fgl + al^) in order. The tunnel mainly passes through high strength pebble soil with lens sand layer in Holocene glacial deposits and alluvium (Q_4_^al^). The boulder volume content in the layer where the tunnel trunk is located is about 23.2%, with poor sortability and some of the pebbles exceeding 50 cm in size. The pebble layer has a strong permeability, and the permeability coefficient is 25-35 m/d. The type of groundwater is phreatic water. The buried depth of the static groundwater level is about 3.80–7.30 m, and the elevation of the stable groundwater level is 532.65–541.25 m. The seismic fortification intensity of the area, where the interzone tunnel is located, is VII degree. The peak acceleration of basic ground motion is 0.10 g, and the characteristic period of basic seismic design is 0.45 s. The geological structure is of simple type, and there are no faults, folds and other geological structures nearby. The geological profile of the evaluated section along the tunnel alignment is presented in Fig. 4.

### Evaluation index selection

**Figure 5:**
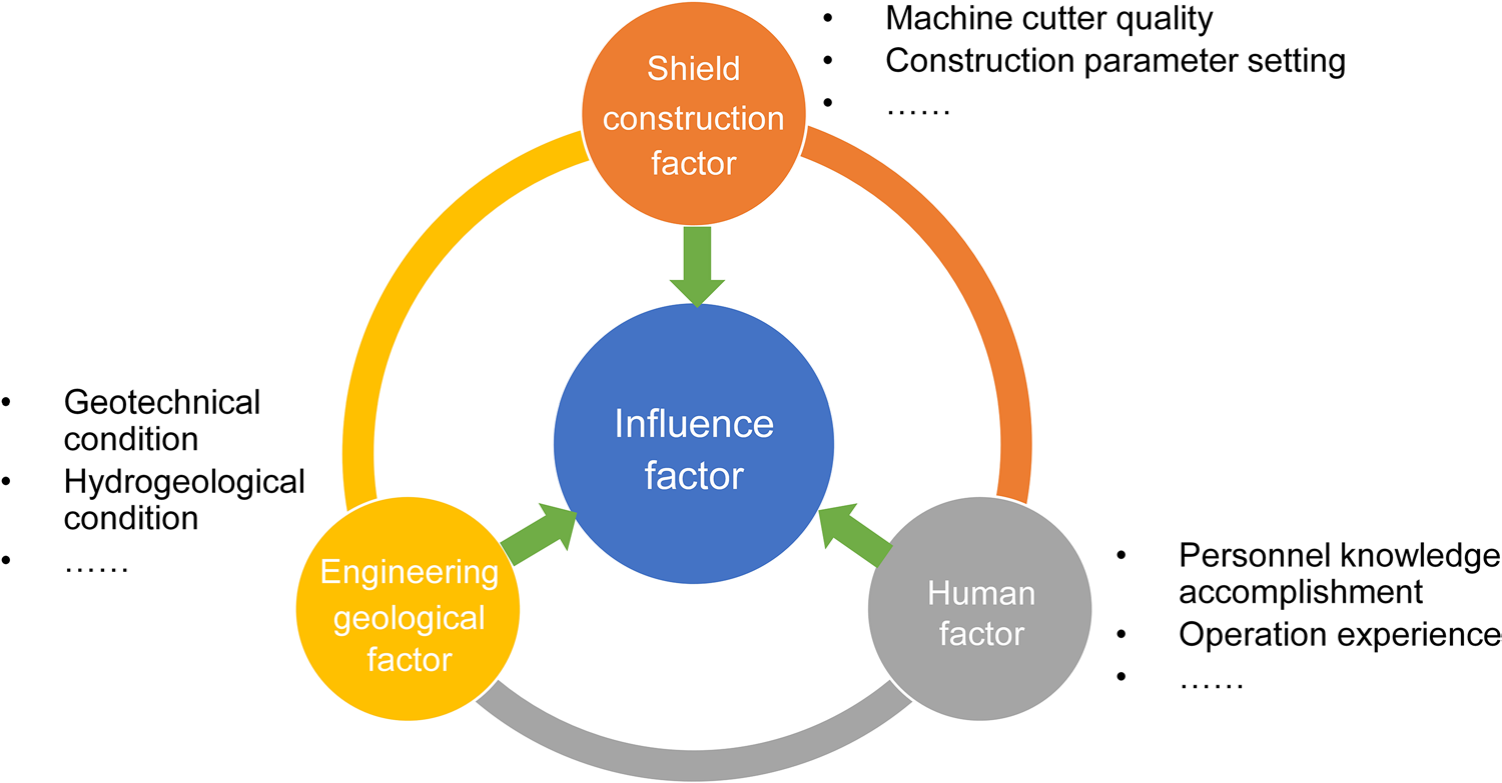
Influence factors of shield tunnel construction safety.

Generally speaking, factors affecting the safety and stability of shield machine construction can be divided into three categories: (1) engineering geological factors; (2) shield construction factors; and (3) human factors^42,43^. Further, engineering geological factors include geotechnical conditions, hydrogeological conditions, etc. Shield construction factors include machine cutter quality, construction parameter settings, etc. Human factors include personnel knowledge accomplishment, operation experience and so on. Figure 5 shows the risk evaluation index system affecting the shield tunnel construction. Among these three categories of influencing factors, the engineering geological conditions of the site are particularly important, due to the potential impact on the others.

**Figure 6:**
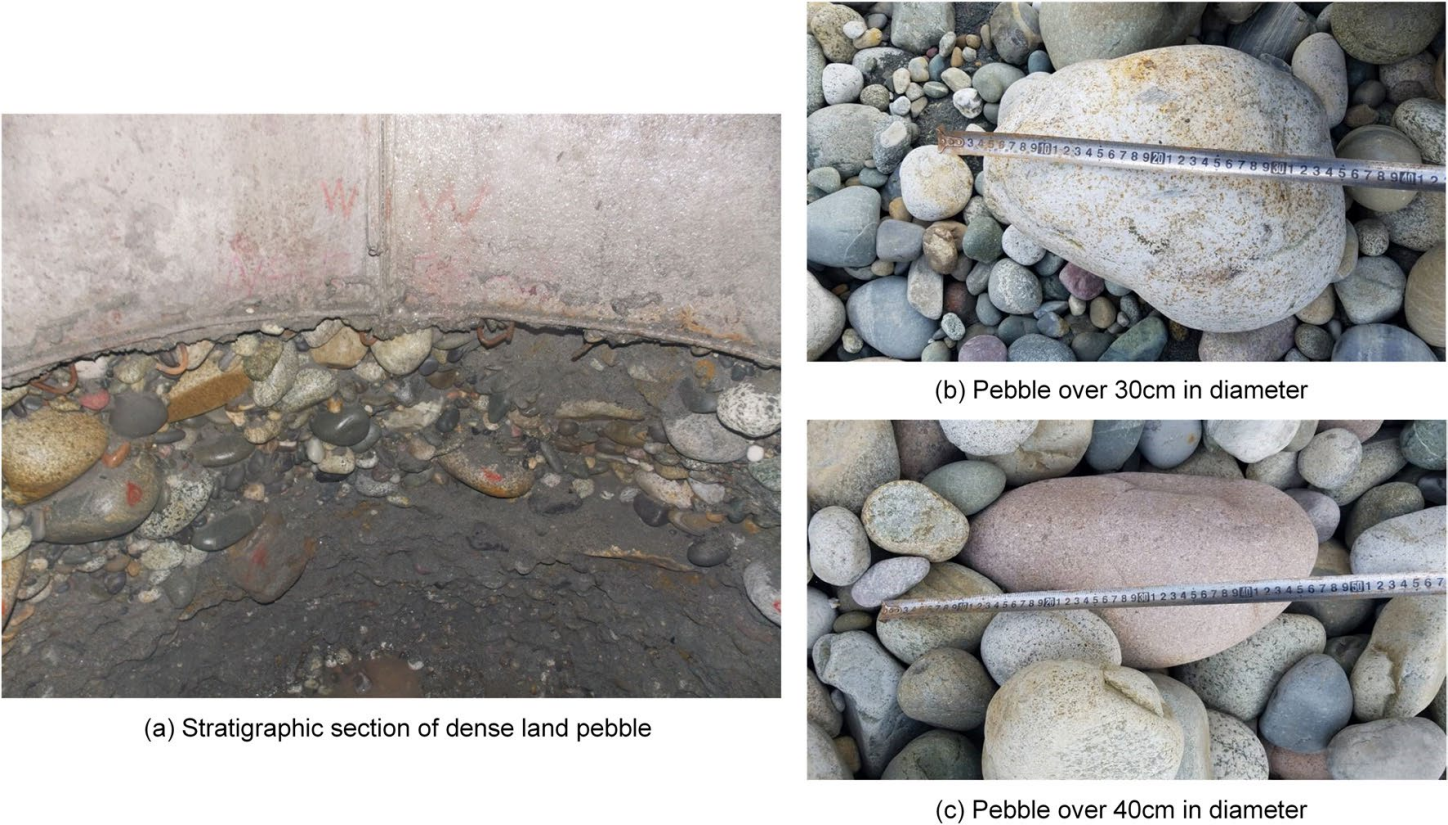
Pebbles and stratigraphic section in the research area.

The tunnel trunk in the study area is basically located in pebble soil, which has poor sort ability and uniformity but high compressive strength. The particle size is mainly concentrated in 20–55 cm, as shown in Fig. 6. The boulders with large particle size tend to stay in the soil compartment, which leads to the difficulty of soil discharge. In the process of shield tunneling, risks such as severe abrasion of shield cutter and failure of shield tunneling are easy to occur. The main structure of the interzone tunnel is basically located below the groundwater. The groundwater mainly consists of pore phreatic water in sandy soil and pebble soil, and the groundwater level along the metro changes greatly with the seasons. Therefore, the water pressure and floating effect of groundwater cannot be ignored. Groundwater and artificially filled soil are slightly corrosive to the steel structure in reinforced concrete, and necessary dewatering measures should be considered before excavation. In the process of reducing groundwater, however, it is prone to flowing sand or piping that will cause uneven ground settlement. What's more, it may also result in settlement deformation or cracking of surrounding existing buildings. The pebble soil layer within the interzone tunnel has good water richness and strong water permeability, which will lead to the seepage, sand gushing and even surface collapse. In addition, factors such as tunnel buried depth, grouting pressure, tunneling speed, etc. will directly affect the surface uplift or subsidence. Combined with the above field survey data and the analysis of the construction design, seven indices are selected to build evaluation index system, including compressive strength of pebble layer, boulder volume content, permeability coefficient, groundwater depth, grouting pressure, tunneling speed and tunnel buried depth.

**Figure 7:**
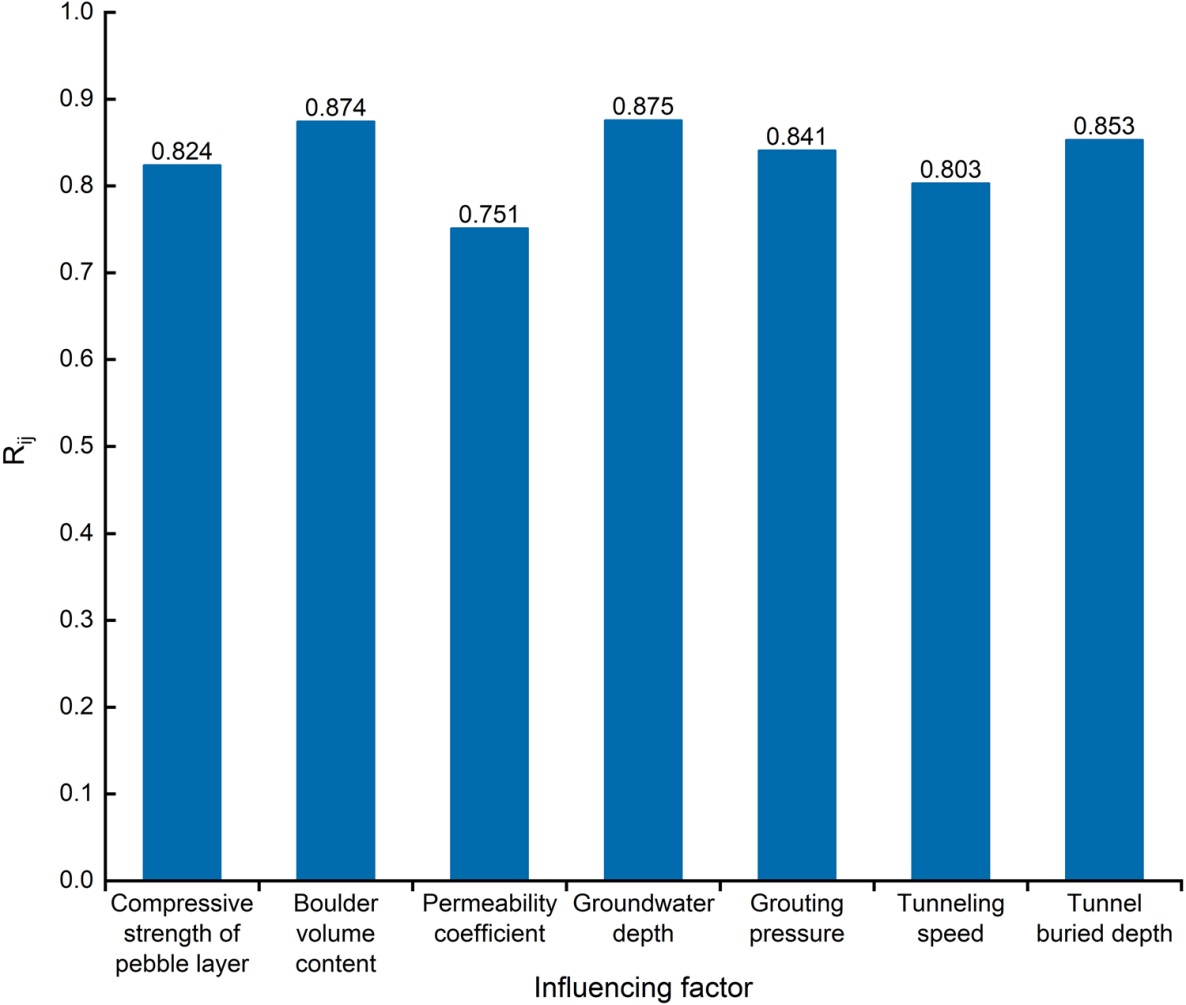
Sensitivity analysis of influencing factors.

Sensitivity analysis of the selected evaluation indices is performed using the cosine amplitude method (CAM). The basic principle of CAM is related to the dot product of cosine functions, i.e., if two vectors are co-linear, the dot product is 1; if two vectors are perpendicular, the dot product is 0. According to the Eq. (22), the relation strength between the input parameter (*I*_*i*_) and output parameter (*I*_*j*_) is obtained, where *N* is the number of datasets. *R*_*ij*_ is in the range of 0–1, with larger values indicating a greater impact^44^. Figure 7 shows the *R*_*ij*_ values of the seven input parameters. The results illustrate that groundwater depth and boulder volume content are the parameters that have the most influence on the risk of shield construction. This is followed by the tunnel buried depth, grouting pressure, compressive strength of pebble layer and tunneling speed, with permeability coefficient being the weakest.

22 $$ R_{ij} = \frac{{\mathop \sum \nolimits_{k = 1}^{N} \left( {I_{ik} I_{jk} } \right)}}{{\sqrt {\mathop \sum \nolimits_{k = 1}^{N} I_{ik}^{2} \mathop \sum \nolimits_{k = 1}^{N} I_{jk}^{2} } }} $$

### Certainty degree

**Figure 8:**
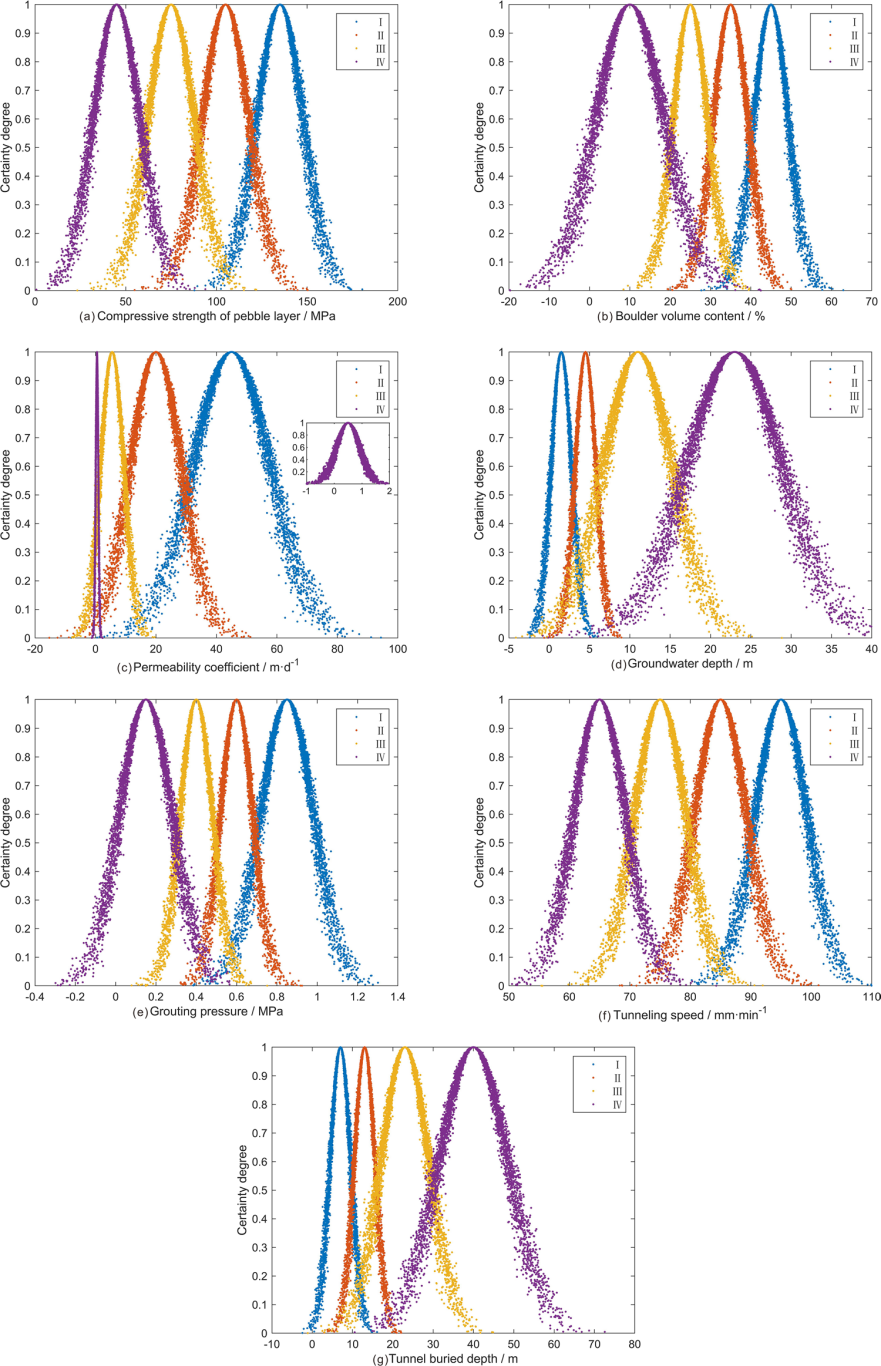
Cloud model diagram of evaluation indices.

The risk degree of shield tunnel construction is divided into I, II, III and IV four grades, corresponding extremely dangerous, highly dangerous, moderately dangerous and lowly dangerous^45,46^. Based on the actual situation of the construction site, previous experience^23,40,47^ and related norms^48,49^, the safety level classification criteria of evaluation indices are established as shown in Table 1. According to the safety level evaluation criteria in Table 1 and Eqs. (1)–(3), the digital eigenvalues (*E*_*x*_*, E*_*n*_*, H*_*e*_) of cloud model and the cloud model evaluation diagram corresponding to each index are obtained, as shown in Table 2 and Fig. 8. Figure 8a–g represent the standard grade cloud model graphs of the seven evaluation indices of compressive strength of pebble layer, boulder volume content, permeability coefficient, groundwater depth, grouting pressure, tunneling speed and tunnel buried depth respectively. Each curve is composed of 5000 cloud droplets, and the distribution range of different indices with different grades can be clearly displayed based on the curves. Different colors represent different security levels and the degree of risk, respectively is blue represents grade I, red represents grade II, orange represents grade III, and purple represents grade IV. The degree of risk decreases gradually from blue to purple. Due to the unclear presentation caused by the large span of the permeability coefficient parameter in different grades, in particular, the grade IV curve is enlarged and presented in the upper right corner of Fig. 8c.

**Table 1:** Safety level evaluation criteria.

Index	I	II	III	IV
Compressive strength of pebble layer (MPa)	> 120	90–120	60–90	< 60
Boulder volume content (%)	> 40	30–40	20–30	< 20
Permeability coefficient (m/d)	> 30	10–30	1–10	< 1
Groundwater depth (m)	< 3	3–6	6–16	> 16
Grouting pressure (MPa)	> 0.7	0.5–0.7	0.3–0.5	< 0.3
Tunneling speed (mm/min)	> 90	80–90	70–80	< 70
Tunnel buried depth (m)	< 10	10–16	16–30	> 30

**Table 2:** Digital eigenvalues of forward cloud model.

Grade	Digital eigenvalues	Index						
		Compressive strength of pebble layer	Boulder volume content	Permeability coefficient	Groundwater depth	Grouting pressure	Tunneling speed	Tunnel buried depth
I	*E*_*x*_	135	45	45	1.5	0.85	95	7
	*E*_*n*_	12.74	4.25	12.74	1.27	0.13	4.25	2.55
	*H*_*e*_	1.274	0.425	1.274	0.127	0.013	0.425	0.255
II	*E*_*x*_	105	35	20	4.5	0.6	85	13
	*E*_*n*_	12.74	4.25	8.49	1.27	0.08	4.25	2.55
	*H*_*e*_	1.274	0.425	0.849	0.127	0.008	0.425	0.255
III	*E*_*x*_	75	25	5.5	11	0.4	75	23
	*E*_*n*_	12.74	4.25	3.82	4.25	0.08	4.25	5.94
	*H*_*e*_	1.274	0.425	0.382	0.425	0.008	0.425	0.594
IV	*E*_*x*_	45	10	0.5	23	0.15	65	40
	*E*_*n*_	12.74	8.49	0.42	5.94	0.13	4.25	8.49
	*H*_*e*_	1.274	0.849	0.042	0.594	0.013	0.425	0.849

Generate a normal random number *x*_*i*_ with expectation of *E*_*x*_ and variance of* E*_*n*_ and a normal random number *y*_*i*_ with expectation of *E*_*n*_ and variance of *H*_*e*_ respectively. According to Eq. (4), calculate the degree of certainty *μ*_*i*_ and input a cloud drop (*x*_*i*_*, μ*_*i*_). Repeat the above steps to generate the required number of cloud drops and make up the cloud. Finally, the cloud model without weighted is formed by the forward normal cloud generator. According to the construction process of shield tunneling machine, the interzone tunnel between Laifeng Road Station and Fengxi Station is divided into 11 tunnel sections with every 150 m. Based on the measured index data of 37 boreholes distributed in 11 mileage sections of the evaluated tunnel section, the average level of measured data of boreholes within each mileage section is compiled, as shown in Table 3. According to the safety level evaluation criteria and the digital eigenvalues of cloud model, the different safety level certainty degrees of each evaluation index of the 11 mileage sections are obtained, as shown in Table 4.

**Table 3:** Field measured data.

Mileage section	Compressive strength of pebble layer (MPa)	Boulder volume content (%)	Permeability coefficient (m/d)	Groundwater depth (m)	Grouting pressure (MPa)	Tunneling speed (mm/min)	Tunnel buried depth (m)
YDK58 + 988.459 ~ YDK59 + 138.459	125.0	21.8	26.9	7.1	0.3389	66.7	9.98
YDK59 + 138.459 ~ YDK59 + 288.459	95.4	29.8	26.9	6.8	0.3936	69.4	10.56
YDK59 + 288.459 ~ YDK59 + 438.459	108.0	32.4	26.9	5.7	0.4924	65.3	11.66
YDK59 + 438.459 ~ YDK59 + 588.459	68.8	20.3	26.9	4.9	0.2039	68.2	12.90
YDK59 + 588.459 ~ YDK59 + 738.459	55.2	15.5	26.9	5.7	0.1826	72.8	14.06
YDK59 + 738.459 ~ YDK59 + 888.459	85.1	18.5	26.9	5.4	0.1565	71.2	15.16
YDK59 + 888.459 ~ YDK60 + 038.459	60.9	14.1	26.9	4.9	0.1307	73.3	16.07
YDK60 + 038.459 ~ YDK60 + 188.459	75.7	16.0	26.9	5.0	0.1254	74.5	17.08
YDK60 + 188.459 ~ YDK60 + 338.459	108.2	21.2	26.9	5.3	0.1290	67.7	18.08
YDK60 + 338.459 ~ YDK60 + 488.459	95.4	13.1	26.9	5.2	0.1758	66.8	19.16
YDK60 + 488.459 ~ YDK60 + 591.691	64.7	13.4	26.9	6.3	0.1742	67.7	19.95

**Table 4:** Certainty degree of different safety levels of each evaluation index.

Mileage section	Safety level	U_x1_	U_x2_	U_x3_	U_x4_	U_x5_	U_x6_	U_x7_
YDK58 + 988.459 ~ YDK59 + 138.459	I	0.587706	0.000525	0.443117	0.005905	0.013360	0.000005	0.494137
	II	0.375027	0.060576	0.601026	0.242818	0.043647	0.007792	0.518281
	III	0.017926	0.626925	0.000326	0.591696	0.629843	0.295944	0.201252
	IV	0.000102	0.470253	0.000001	0.112966	0.438334	0.657528	0.028056
YDK59 + 138.459 ~ YDK59 + 288.459	I	0.065152	0.029885	0.443117	0.009231	0.029063	0.000050	0.423576
	II	0.618381	0.488345	0.601026	0.302632	0.125757	0.027744	0.581677
	III	0.376038	0.482651	0.000326	0.572283	0.713790	0.484111	0.224025
	IV	0.013834	0.202987	0.000001	0.105331	0.313039	0.509159	0.031813
YDK59 + 288.459 ~ YDK59 + 438.459	I	0.222817	0.078398	0.443117	0.043114	0.097606	0.000001	0.294961
	II	0.690192	0.651384	0.601026	0.543670	0.451949	0.003668	0.673849
	III	0.142120	0.311114	0.000326	0.494626	0.487917	0.211068	0.271073
	IV	0.001982	0.138477	0.000001	0.080511	0.136294	0.697190	0.040110
YDK59 + 438.459 ~ YDK59 + 588.459	I	0.000752	0.000203	0.443117	0.112968	0.001428	0.000019	0.177228
	II	0.092821	0.033869	0.601026	0.666374	0.002680	0.016242	0.711364
	III	0.663068	0.538828	0.000326	0.434619	0.161019	0.399340	0.329372
	IV	0.288577	0.521822	0.000001	0.065421	0.688290	0.583700	0.051536
YDK59 + 588.459 ~ YDK59 + 738.459	I	0.000024	0.000006	0.443117	0.043114	0.000962	0.000619	0.101071
	II	0.015155	0.003422	0.601026	0.543670	0.001866	0.099565	0.671466
	III	0.392800	0.220049	0.000326	0.494626	0.113818	0.667343	0.387577
	IV	0.589251	0.656772	0.000001	0.080511	0.702607	0.281585	0.064481
YDK59 + 738.459 ~ YDK59 + 888.459	I	0.016205	0.000059	0.443117	0.063101	0.000583	0.000198	0.055620
	II	0.385093	0.015541	0.601026	0.599976	0.001169	0.056981	0.576196
	III	0.596754	0.414031	0.000326	0.472293	0.071126	0.597565	0.444345
	IV	0.056985	0.579191	0.000001	0.074572	0.707121	0.386868	0.078998
YDK59 + 888.459 ~ YDK60 + 038.459	I	0.000111	0.000002	0.443117	0.112968	0.000348	0.000867	0.032511
	II	0.034980	0.001531	0.601026	0.666374	0.000692	0.116712	0.472742
	III	0.523864	0.150861	0.000326	0.434619	0.042697	0.680943	0.491069
	IV	0.468259	0.682514	0.000001	0.065421	0.697430	0.251185	0.092795
YDK60 + 038.459 ~ YDK60 + 188.459	I	0.003174	0.000009	0.443117	0.101120	0.000313	0.001875	0.017125
	II	0.186328	0.004491	0.601026	0.656427	0.000615	0.165892	0.353212
	III	0.698153	0.248609	0.000326	0.442176	0.038243	0.695644	0.541074
	IV	0.160857	0.645806	0.000001	0.067178	0.693755	0.185199	0.110124
YDK60 + 188.459 ~ YDK60 + 338.459	I	0.226379	0.000362	0.443117	0.033069	0.000336	0.000012	0.008627
	II	0.688649	0.048351	0.601026	0.641670	0.000667	0.012819	0.246972
	III	0.139415	0.594553	0.000326	0.443170	0.041223	0.364017	0.586965
	IV	0.001920	0.491174	0.000001	0.084663	0.696312	0.611236	0.129463
YDK60 + 338.459 ~ YDK60 + 488.459	I	0.065152	0.000001	0.443117	0.080321	0.000846	0.000006	0.003849
	II	0.618381	0.000830	0.601026	0.631309	0.001659	0.008202	0.156177
	III	0.376038	0.111517	0.000326	0.457267	0.101167	0.302528	0.630487
	IV	0.013834	0.696018	0.000001	0.070801	0.705182	0.653501	0.152861
YDK60 + 488.459 ~ YDK60 + 591.691	I	0.000289	0.000001	0.443117	0.019022	0.000820	0.000012	0.002024
	II	0.057367	0.001001	0.601026	0.412472	0.001613	0.012819	0.106733
	III	0.600764	0.122448	0.000326	0.538041	0.098353	0.364017	0.657215
	IV	0.380164	0.692417	0.000001	0.093442	0.705645	0.611236	0.171643

### Comprehensive weight

**Figure 9:**
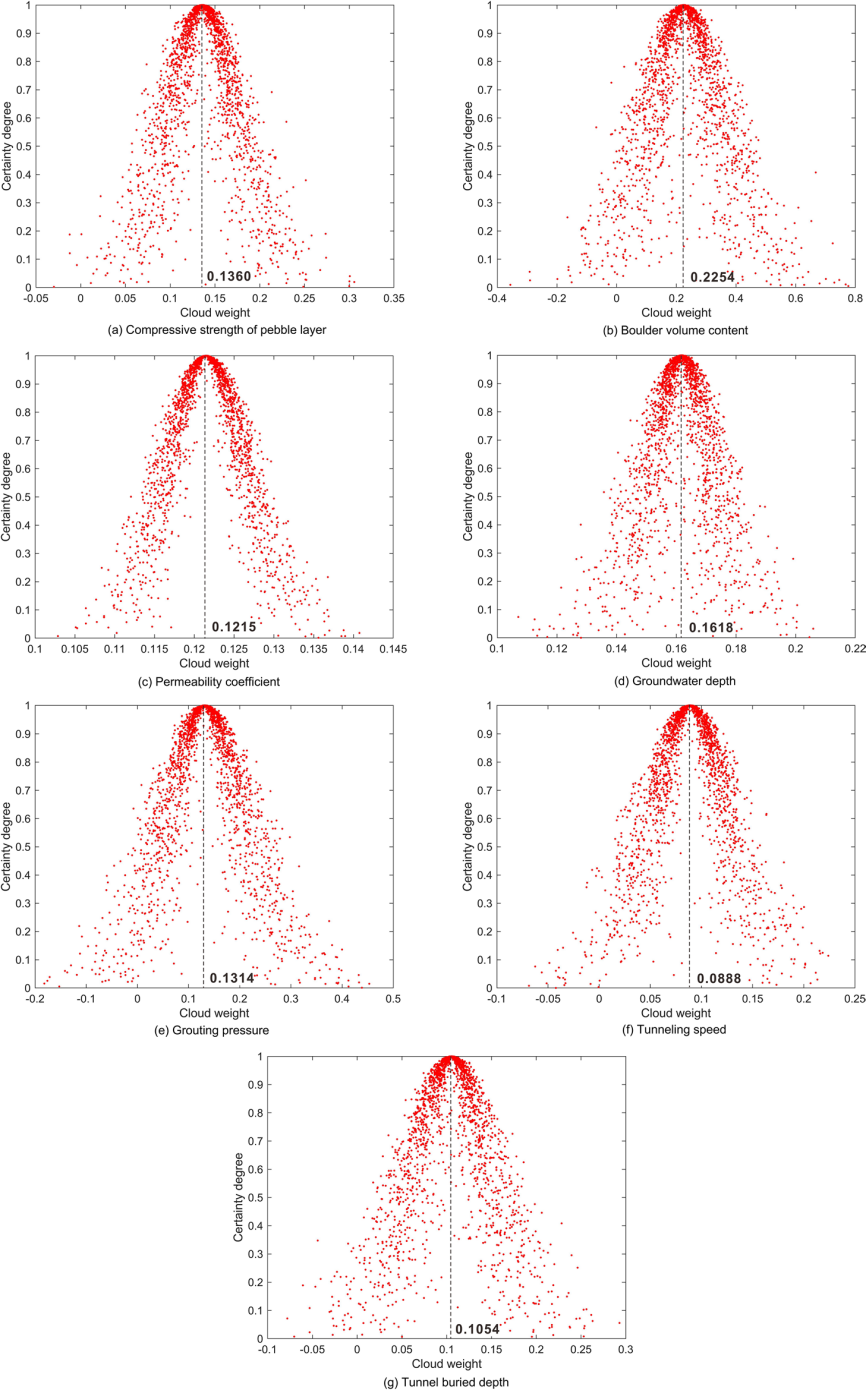
Cloud weight of evaluation indices.

**Figure 10:**
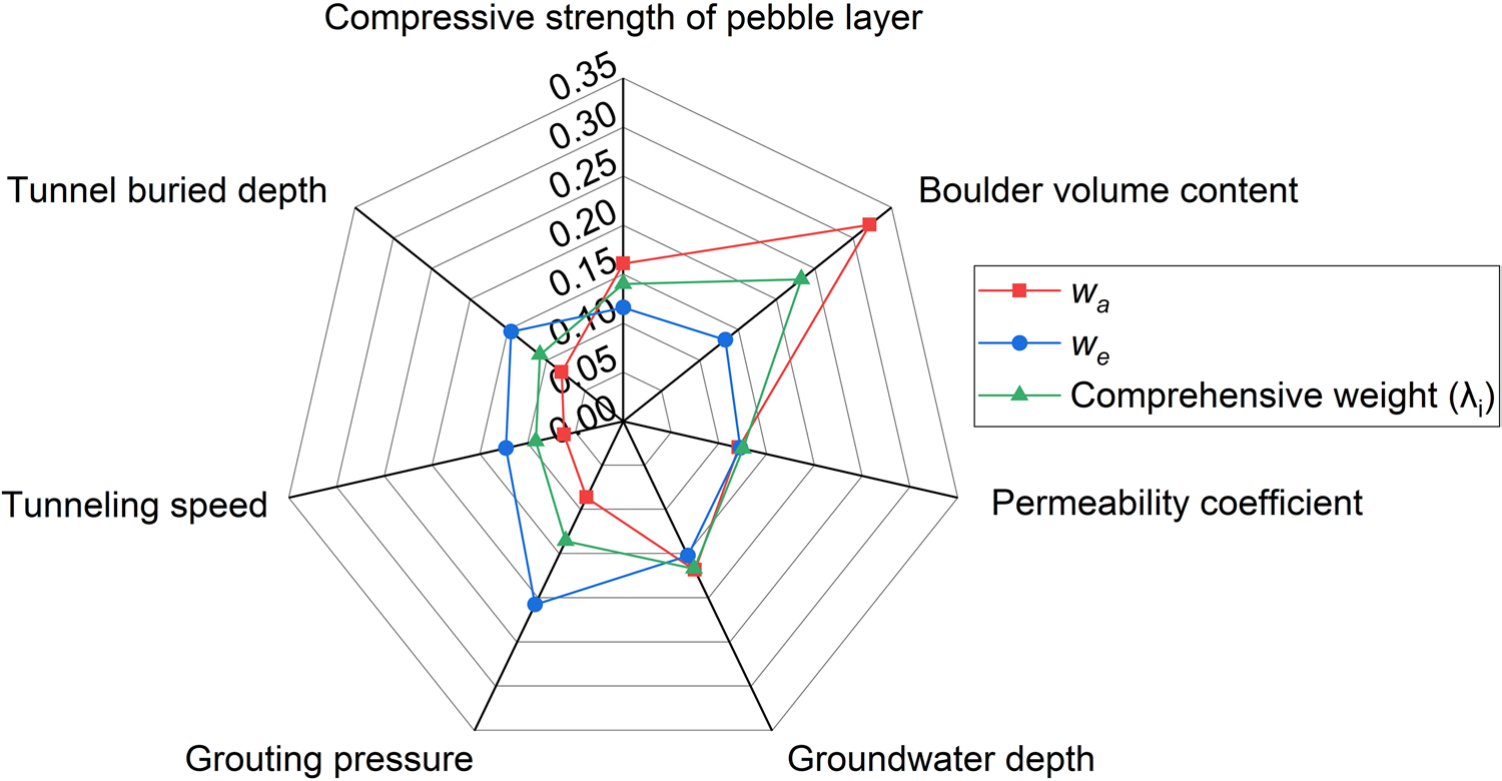
Assignment and comparison of evaluation index weights.

The reasonable assignment of comprehensive weights of evaluation indices has a great impact on the accuracy of the assessment results. On the one hand, compare the importance of the seven evaluation indices based on AHP, including pebble layer compressive strength, boulder volume content, permeability coefficient, groundwater depth, grouting pressure, tunneling speed and tunnel buried depth. The subjective weights (*w*_*a*_) can be calculated by using Eqs. (9)–(15), as shown in Table 5. On the other hand, combined with the site measured index data in Table 3 and Eqs. (16)–(19), the information entropy (*E*_*j*_) and objective weights (*w*_*e*_) of seven indices can be obtained by using EWM, as shown in Table 6. The subjective weights and objective weights obtained by AHP and EWM are introduced into the reverse cloud model as initial data to solve the comprehensive weights. These weights are regarded as two groups of cloud droplets surrounding the true weights. According to Eqs. (5)–(8), the reverse normal cloud generator is used to solve the corresponding digital eigenvalues of the clouds that automatically formed by randomly generated several cloud droplets. Figure 9a–g display the generation results of each random cloud droplet that constitutes the comprehensive weight cloud for each of the seven evaluation indices respectively, where the horizontal coordinate is the weight and the vertical coordinate is the certainty degree. The expectation of the composed cloud is the requested cloud weight, corresponding to the value of the horizontal coordinate at the peak location of the certainty degree. Then, Eq. (20) is used to normalize the composite cloud weights, and the comprehensive weights of evaluation indices are presented in Table 7. Figure 10 displays visually the assignment and comparison of subjective weights, objective weights and comprehensive weights of evaluation indices. As can be seen, the comprehensive weight has great rationality by integrating the effects of subjective and objective weights. The empirical assignment of subjective and objective weights in the traditional combination weighting method is improved by the reverse cloud model^50^.

**Table 5:** Subjective weights of evaluation indices.

Index	*w*_*a*_
Compressive strength of pebble layer	0.1608
Boulder volume content	0.3215
Permeability coefficient	0.1206
Groundwater depth	0.1683
Grouting pressure	0.0864
Tunneling speed	0.0619
Tunnel buried depth	0.0804

**Table 6:** Objective weights of evaluation indices.

Index	*E*_*j*_	*w*_*e*_
Compressive strength of Pebble layer	0.8413	0.1161
Boulder volume content	0.8179	0.1332
Permeability coefficient	0.8326	0.1224
Groundwater depth	0.7920	0.1521
Grouting pressure	0.7165	0.2073
Tunneling speed	0.8324	0.1226
Tunnel buried depth	0.7997	0.1464

**Table 7:** Comprehensive weights of evaluation indices.

Index	*w*_*a*_	*w*_*e*_	Cloud weight	Comprehensive weight
Compressive strength of pebble layer	0.1608	0.1161	0.1360	0.1402
Boulder volume content	0.3215	0.1332	0.2254	0.2323
Permeability coefficient	0.1206	0.1224	0.1215	0.1252
Groundwater depth	0.1683	0.1521	0.1618	0.1668
Grouting pressure	0.0864	0.2073	0.1314	0.1354
Tunneling speed	0.0619	0.1226	0.0888	0.0915
Tunnel buried depth	0.0804	0.1464	0.1054	0.1086

### Results

**Figure 11:**
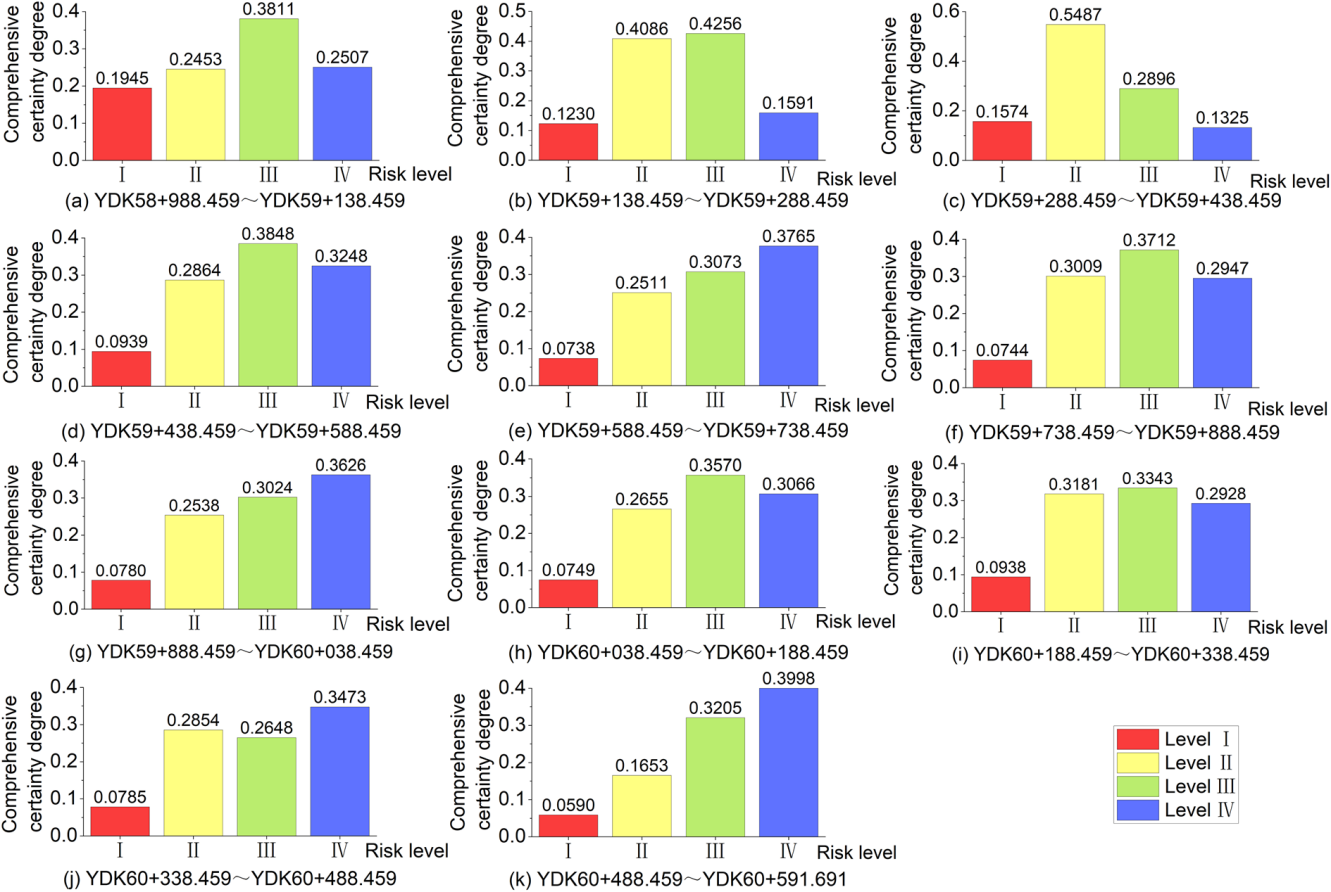
Comprehensive certainty degree distribution of different safety levels in each mileage section.

Combined with the initial certainty degree and index weight, Eq. (21) is used to calculate the comprehensive certainty degree of each evaluation index. The comprehensive certainty degree of each safety level in each mileage section can be obtained by adding the certainty degree of different evaluation indices of each safety level, as shown in Table 8. Figure 11a–k present the distribution of risk level certainty within each of the 11 mileage sections, where the horizontal coordinates indicate the different safety levels and the vertical coordinates indicate the degree of comprehensive certainty. The evaluation results can be determined according to the principle of maximum certainty. The risk level of 11 mileage sections can be visualized in Fig. 11 as III, III, II, III, IV, III, IV, IV, III, III, IV, IV.

**Table 8:** Comprehensive certainty of different safety levels of each mileage section.

Mileage section	Comprehensive certainty				Safety level
	U_1_	U_2_	U_3_	U_4_	
YDK58 + 988.459 ~ YDK59 + 138.459	0.1945	0.2453	0.3811	0.2507	III
YDK59 + 138.459 ~ YDK59 + 288.459	0.1230	0.4086	0.4256	0.1591	III
YDK59 + 288.459 ~ YDK59 + 438.459	0.1574	0.5487	0.2896	0.1325	II
YDK59 + 438.459 ~ YDK59 + 588.459	0.0939	0.2864	0.3848	0.3248	III
YDK59 + 588.459 ~ YDK59 + 738.459	0.0738	0.2511	0.3073	0.3765	IV
YDK59 + 738.459 ~ YDK59 + 888.459	0.0744	0.3009	0.3712	0.2947	III
YDK59 + 888.459 ~ YDK60 + 038.459	0.0780	0.2538	0.3024	0.3626	IV
YDK60 + 038.459 ~ YDK60 + 188.459	0.0749	0.2655	0.3570	0.3066	III
YDK60 + 188.459 ~ YDK60 + 338.459	0.0938	0.3181	0.3343	0.2928	III
YDK60 + 338.459 ~ YDK60 + 488.459	0.0785	0.2854	0.2648	0.3473	IV
YDK60 + 488.459 ~ YDK60 + 591.691	0.0590	0.1653	0.3205	0.3998	IV

## Discussion

During the shield tunneling process, the initial equilibrium state of the strata is destroyed by the jacking, squeezing and shear friction of the shield shell. The soil particles shift, resulting in the redistribution of ground stress and surface subsidence eventually. Especially when shield machine tunneling in water-rich sandy pebble strata with large particle size, the accident of ground collapse is highly likely to occur. Controlling the surface deformation and defining the monitoring level of project are important means to ensure the safety and stability of construction. Furthermore, the engineering monitoring level is closely related to the construction safety level. In other words, the degree of ground settlement can be used as a characterization of the construction risk level. The range of surface settlement corresponding to different safety levels are obtained by referring to the code for monitoring measurement of urban rail transit engineering, as shown in Table 9^51^. In order to justify the assessment results of Chengdu metro engineering case in the previous chapter, the surface settlement values were monitored to count the average surface settlement values caused by construction. And then, the theoretical safety levels of each mileage section were delineated and compared with the evaluation safety level, as shown in Table 10.

**Table 9:** Range of ground surface settlement for different safety levels.

Safety level	Degree of risk	Surface settlement range (mm)
I	Extremely dangerous	36–50
II	Highly dangerous	30–36
III	Moderately dangerous	24–30
IV	Lowly dangerous	0–24

**Table 10:** Average surface settlement and theoretical safety level.

Mileage section	Average surface settlement (mm)	Theoretical safety level	Evaluation Safety level
YDK58 + 988.459 ~ YDK59 + 138.459	25.74	III	III
YDK59 + 138.459 ~ YDK59 + 288.459	28.92	III	III
YDK59 + 288.459 ~ YDK59 + 438.459	38.71	I	II
YDK59 + 438.459 ~ YDK59 + 588.459	24.92	III	III
YDK59 + 588.459 ~ YDK59 + 738.459	22.45	IV	IV
YDK59 + 738.459 ~ YDK59 + 888.459	28.25	III	III
YDK59 + 888.459 ~ YDK60 + 038.459	15.27	IV	IV
YDK60 + 038.459 ~ YDK60 + 188.459	26.54	III	III
YDK60 + 188.459 ~ YDK60 + 338.459	31.35	II	III
YDK60 + 338.459 ~ YDK60 + 488.459	18.64	IV	IV
YDK60 + 488.459 ~ YDK60 + 591.691	17.36	IV	IV

**Figure 12:**
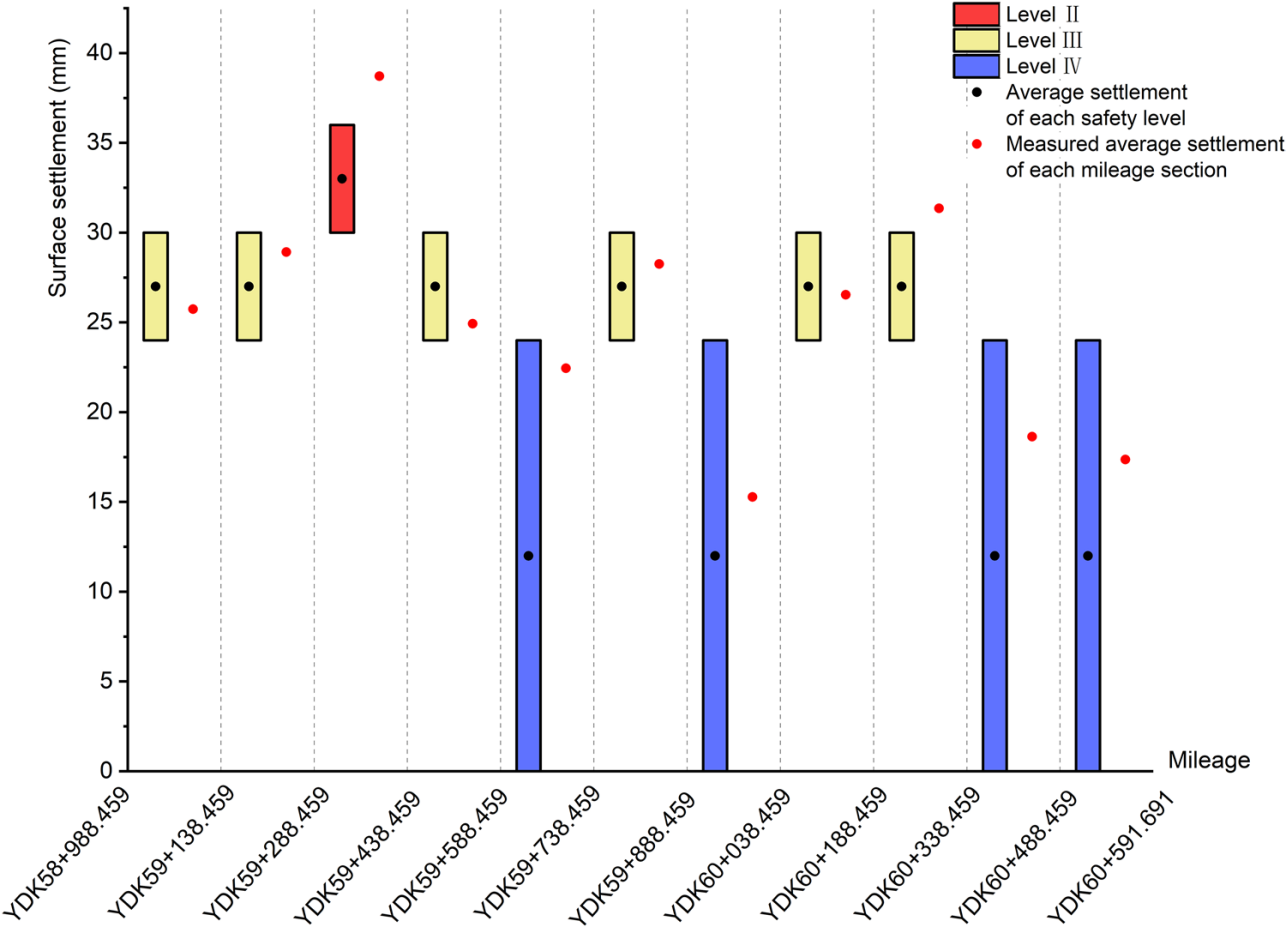
Comparison of evaluation results with the measured average surface settlement.

Figure 12 visually shows the difference between the actual settlement value and the surface subsidence range corresponding to the evaluation safety level. The abscissa corresponds to different mileage, and the interval corresponds to the mileage section. The ordinate corresponds to the surface settlement value. The red, yellow and blue rectangles represent the predicted settlement ranges with safety levels II, III and IV respectively. The black dots are the median of the predicted range of surface subsidence, and the red dots represent the actual average settlement value. As shown in Fig. 12, 9 out of 11 the evaluation safety levels of different mileage sections are in accord with the reality. There is just a little difference between the predicted results and the actual settlement situation of the remaining 2 mileage sections. The maximum average settlement displacement in the construction section of shield tunnel is 38.71 mm and most of the settlement displacement at other mileages are more than 24 mm. The safety level of YDK58 + 988.459 ~ YDK59 + 588.459 mileage section is basically II and III, in a relatively dangerous state. The overall surface settlement is large, which may be caused by the high boulder volume content and shallow tunnel buried depth. With the increasing buried depth of the shield tunnel and decreasing boulder volume content, the overall surface settlement in the mileage section from YDK59 + 588.459 to YDK60 + 591.691 has significantly decreased and the risk level is more in the direction of III and IV. Among them, there is an abrupt increase of surface settlement in the mileage sections YDK59 + 738.459 ~ YDK59 + 888.459 and YDK60 + 038.459 ~ YDK60 + 338.459, this may be due to the high compressive strength of pebble layer. Based on the above results, it is worth mentioning that the general trend of settlement, which is in the whole shield tunnel construction section from the Laifeng Road Station to Fengxi Station, is consistent with reality. Hence, the feasibility and accuracy of the whole evaluation process composed of cloud model, AHP and EWM for shield tunnel construction risk assessment in the water-rich sandy pebble strata of Chengdu Metro can be verified.

## Conclusions

This paper presented an evaluation model to assess the shield tunnel construction risk in the large particle size water-rich sandy pebble strata. In this method, cloud model, AHP and EWM are used to solve the membership of uncertain parameters and to assign indicator weights. With Chengdu Metro project in China as an engineering case study, the model is applied to evaluate the construction safety level and verified by the field measured data. The following conclusions can be drawn from the above work.

1. The proposed model integrates both engineering geological conditions and shield construction parameters. The index evaluation system for shield construction risk in large particle size water-rich sandy pebble strata is established, including compressive strength of pebble layer, boulder volume content, permeability coefficient, groundwater depth, grouting pressure, tunneling speed and tunnel buried depth. The model can ensure the accuracy of evaluation results, while maximizing the use of relevant data and reducing workload.
2. The proposed method introduces the reverse cloud model to the index weight processing of risk assessment, which solves the defect that the existing weight setting method cannot reflect the cognitive uncertainty. Combined with AHP and EWM, both subjectivity and objectivity are taken into account. This method not only ensures the rationality of index weighting, but also realizes the gradual optimization of the weight solving process.
3. This paper enriches the risk assessment study of shield tunnel construction in large particle size water-rich sandy pebble strata, different from the common sandy or clay soils. The results provide insights into the establishment of risk evaluation system for similar engineering projects, and help to predict and avoid risk in advance.

## Data Availability

All data generated or analysed during this study are included in this published article.

## Abbreviations

*a*: Minimum boundary value of index grade evaluation
*b*: Maximum boundary value of index grade evaluation
**B**: Optimal transfer matrix
**C**: Consistency judgment matrix
*E*_*j*_: Information entropy
*E*_*n*_ (*E*_*n*_^*’*^): Entropy of forward (reverse) cloud model
*E*_*x*_ (*E*_*x*_^*’*^): Expectation of forward (reverse) cloud model
*H*_*e*_ (*H*_*e*_^*’*^): Hyper entropy of forward (reverse) cloud model
*i* and *j*: Serial number of evaluation index
*m*: Number of samples
*n*: Number of evaluation index
**R**: Importance judgment matrix
*R*_*ij*_: Relation strength
*S*^*2*^: Sample variance
*U*_*x*_: Comprehensive certainty degree
*w*_*a*_: Subjective weight
*w*_*e*_: Objective weight
*w*_*i*_: Composite cloud weight
**X**: Matrix of sample data values
*x*_*i*_: Normal random number with expectation *E*_*x*_ and variance *E*_*n*_
*y*_*i*_: Normal random number with expectation *E*_*n*_ and variance *H*_*e*_
*λ*_*i*_: Comprehensive weight
*μ*_*i*_: Certainty degree
